# Effects of High Hydrostatic Pressure on Water Absorption of Adzuki Beans 

**DOI:** 10.3390/foods4020148

**Published:** 2015-05-14

**Authors:** Shigeaki Ueno, Toru Shigematsu, Mineko Karo, Mayumi Hayashi, Tomoyuki Fujii

**Affiliations:** 1 Faculty of Education, Saitama University, 255 Shimo Okubo, Sakura-ku, Saitama 338-8570, Japan; 2 Faculty of Applied Life Sciences, Niigata University of Pharmacy and Applied Life Sciences, 265-1 Higashijima, Akiha-ku, Niigata 956-8603, Japan; E-Mails: shige@nupals.ac.jp (T.S.); karo@nupals.ac.jp (M.K.); mhayashi@nupals.ac.jp (M.H.); 3 Graduate School of Agricultural Science, Tohoku University, 1-1Tsutsumidori-Amamiyamachi, Aoba-ku, Sendai 981-8555, Japan; E-Mail: atomic@bios.tohoku.ac.jp

**Keywords:** high hydrostatic pressure, beans, water absorption, kinetic analysis

## Abstract

The effect of high hydrostatic pressure (HHP) treatment on dried soybean, adzuki bean, and kintoki kidney bean, which are low-moisture-content cellular biological materials, was investigated from the viewpoint of water absorption. The samples were vacuum-packed with distilled water and pressurized at 200 MPa and 25 °C for 10 min. After the HHP treatment, time courses of the moisture contents of the samples were measured, and the dimensionless moisture contents were estimated. Water absorption in the case of soybean could be fitted well by a simple water diffusion model. High pressures were found to have negligible effects on water absorption into the cotyledon of soybean and kintoki kidney bean. A non-linear least square method based on the Weibull equation was applied for the adzuki beans, and the effective water diffusion coefficient was found to increase significantly from 8.6 × 10^−13^ to 6.7 × 10^−10^ m^2^/s after HHP treatment. Approximately 30% of the testa of the adzuki bean was damaged upon HHP treatment, which was comparable to the surface area of the testa in the partially peeled adzuki bean sample. Thus, HHP was confirmed to promote mass transfer to the cotyledon of legumes with a tight testa.

## 1. Introduction

Traditionally, rice—an important source of carbohydrates—is the most widely consumed staple food in Japan, but recently, there has been a marked increase in the consumption of functional foods. Examples of such functional foods include polyphenol-enriched adzuki bean, peptide-/GABA-enriched soybean, and white kidney bean (rich in minerals, vitamins, and fiber). These beans are traditionally cooked in various forms in Japan; for example, adzuki beans are combined with white rice to prepare red rice; fermented soybeans are used to prepare *Natto*; and *Cereal coarse Miso* (fermented bean paste) is a coarse mixture of soybean, adzuki beans, barely, brown rice, and oats. Although such coarse cereal mixtures are popular, cooking them is time-consuming because of the difference in the water absorption behavior of the constituent grains. Uniform/homogeneous water absorption will aid the effective cooking of these beans and increase the demand for such functional foods.

Water soaking is typically used as the pretreatment method for beans to remove water-soluble substances and ensure effective cooking. Generally, for ease of cooking and processing, beans are soaked in water to allow them to swell so that the cooking time can be reduced [1]. Several researchers have attempted to study the water absorption behavior of various types of beans. Volume changes in soybeans, adzuki beans, and kidney beans were determined from the particle density and bulk density [1]. The bulk densities and specific bulk volumes of the beans were found to be quadratic functions of moisture content. The water absorption behavior of adzuki beans and soybeans was examined over the temperature range 10–60 °C, taking into account the initial water absorption period and the falling rate period in the overall water absorption process [2]. However, hardly any research has been conducted on water absorption in high hydrostatic pressure (HHP)-treated adzuki beans.

High hydrostatic pressure (HHP) treatment, which is a non-thermal method, is regarded with special interest in the food industry, as it can help in reducing the cooking time without affecting the nutritional value of the food. However, high-pressure treatment has several disadvantages. Ueno *et al*. reported that high pressures of 200 and 400 MPa cause structural changes in *Brassica rapa* root and onion, leading to partial and total destruction of the cell structure [3]. Further, high-pressure treatment above 100 MPa results in lipid-phase transition, which in turn destroys the internal structure and membrane structure of foodstuff, accelerates mass transfer, alters the texture of the food, and degrades the physicochemical properties [4,5,6]. High-pressure-induced damages to cellular structure were also elucidated in a separate study on the relative drying rate (RDR) of Japanese radish samples before and after HHP pretreatment; the RDR was found to be similar to that after chloroform vapor treatment but lower than that in the case of heat treatment and freeze-thaw pretreatment [7]. HHP on legumes such as green bean [8,9] chickpea [10], cowpea [11], and soybean [12,13] had been reported from the viewpoints of structural, physical or biochemical changes. However, in another study, Eshtiaghi *et al.* reported that high-pressure treatment on green bean resulted in incomplete rehydration and helped retain the texture [8]. 

In this study, considering the potential of HHP in inducing beneficial structural changes in food products, we decided to investigate the effect of high-pressure treatment on dried beans with low moisture contents. Increased water uptake in treated beans can help in converting them into valuable food products.

## 2. Materials and Methods 

### 2.1. Theoretical Methods

Methods used to determine the moisture content of the control and treated beans follow the theoretical considerations described in earlier reports [14,15]. The infinite series diffusion equation was used to model the water absorption process. The dimensionless moisture content can be written as follows:

*M_t_* = (m*_t_* − m_e_)/(m_0_ − m_e_) = (6/π^2^)･exp[(π^2^*Dθ*)/*R*^2^] (1)

where *M_t_* is the dimensionless moisture content (-), m*_t_* is the moisture content (g-water/g-dray basis) at time *t* (s), m_e_ is the final saturated water content (g-water/g-dry basis), and m_0_ is the initial moisture content (g-water/g-dry basis) at time zero. Additionally, *M_t_* the dimensionless moisture content was converted as Equation (1) with diffusion coefficient *D* (m^2^ s^−1^), time of water absorption *θ* (s), and radius of particle *R* (m). Using the measured moisture content, a non-linear regression method was applied to an approximate solution of the diffusion equation:

*M_t_* = A_1_exp(−*K_1_t*) + *M_e_* (2)

where *M_t_* is the moisture content, A_1_ is a constant, *K_1_* is the absorption rate constant (h^−1^), *t* is the absorption time (h) and *M_e_* is the apparent initial moisture content (-).

*M_R_* = A_R1_exp(−*K_R1_t*) (3)

where *M_R_* is the dimensionless moisture content, A_R1_ is a constant, *K_R1_* is the absorption rate constant of dimensionless moisture content (s^−1^), and *t* is the absorption time (s). 

Thus, the appropriate model could be estimated from the experimental values evaluated by applying a non-linear regression procedure to Equation (2). In a particular case where the water absorption rate had a range, the simple diffusion equation was replaced by the Weibull equation:

*M_t_* = A_2_exp(−*K*_2_*t*^n^) + *M_e_* (4)

where *M_t_* is the moisture content, A_2_ is a constant, *K_2_* is the absorption rate constant (s^−1^), *t* is the absorption time (s), n is a constant and *M_e_* is the apparent initial moisture content (-).

*M_R_* = A_R2_exp(−*K_R_*_2_*t*^n^) (5)

where *M_R_* is the dimensionless moisture content, A_R2_ is a constant, *K_R2_* is the absorption rate constant of dimension less moisture content (s^−1^), and *t* is the absorption time (s), n is a constant.

The effective diffusion coefficients of the samples were calculated by following equation:

*K* = (*Dπ^2^*)/*l*^2^ (6)

where *K* is the absorption rate constant of dimension less moisture content (s^−1^). *D* is the effective diffusion coefficient (m^2^ s^−1^), and *l* is the sample size (m). Since the beans we considered are not spherical in shape, radius cannot be considered as an adequate estimate of the sample size. Hence, the harmonic average of sample length was calculated by

*l* = 1/((1/*L_l_*) + (1/*L_m_*)) (7)

where *l* is the harmonic average of the size, *L_l_* is the major length, and *L_m_* is the minor length of the sample (m).

### 2.2. Sample Preparation

**Figure 1:**
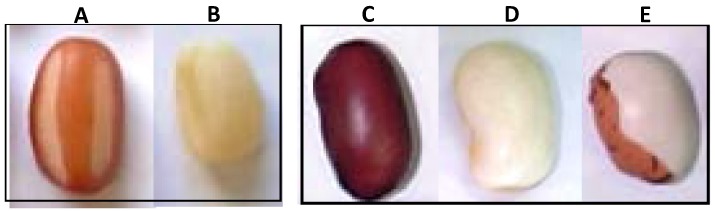
Photographs of partially peeled adzuki bean (**A**), completely peeled adzuki bean (**B**), kintoki kidney bean (**C**), white kidney bean (**D**), and toramame kidney bean (**E**).

Dried beans were chosen as the low-moisture cellular material model. Soybeans (*Glycine max* (L.) Merr.) were harvested from Niigata, while adzuki beans (*Vigna angularis* (Wild.) Ohwi et Ohashi), *kintoki* kidney beans (*Phaseolus vulgaris* L.), white *ingen* kidney beans (*Phaseolus vulgaris* L.), and *toramame* kidney beans (*Phaseolus vulgaris* L.) harvested in Hokkaido were purchased from a local market in Niigata. The testa of the adzuki beans was partially or completely peeled off with a metallic file. The average area ratio of the testa surface in the partially peeled adzuki beans was 31% ± 4.7% (Figure 1).

Approximately 50 g of beans were soaked in distilled water (threefold volume) in a polyethylene bag (Mekkin Kensa Bag, Eiken kizai, Tokyo, Japan) and vacuum-sealed using an industrial sealer (TM-HG, Furukawa MFG Co. Ltd., Tokyo). All tests were carried out in duplicate.

### 2.3. High-Pressure Pretreatment

Samples were packed under vacuum and soaked in hydraulic fluid (distilled water) in a high-hydrostatic pressure vessel (inner diameter 60 mm, depth 180 mm) that could withstand a maximum pressure of 686 MPa (WIP, Kobe Steel, Kobe, Japan). The samples were pressurized at 200 MPa for 10 min at 20 °C. The pressure increase rate was 3.0 to 3.7 MPa/s; decompression was completed within 40 s. The temperature of distilled water in the high-pressure vessel varied within 2 °C during the pressure treatment. Some research groups have reported that HHP over 200 MPa produced important structural changes in proteins of legumes, and which led to the denaturation of proteins concerning with biochemical reactions and loss of nutritious values [16,17]. We therefore evaluated after treating at 200 MPa to minimize any loss of biochemical changes.

### 2.4. Water Absorption Test 

After the treatment, the samples were washed with water and blotted with paper towels. The samples were then placed in a textile mesh pack (Ocha-Pack M, KOMERI, Niigata, Japan) and allowed to soak in distilled water at 25 °C until all the water was absorbed. The treated and untreated samples were soaked under identical conditions. Finally, the samples were removed from the beaker and blotted with paper towels. The final weights (*W_t_*) of the samples were then recorded. 

### 2.5. Drying Test 

The initial moisture content of the beans was measured by a drying test. Each bean sample was dried in a circular drier (SSV-51EY, Ikeda Rika, Tokyo) at 110 °C for 48 h, and the initial weight *W*_0_ was recorded. The moisture content *m_t_* of the samples (g-water/g- dray basis) was calculated using the equation

*m_t_* = (*W_t_* − *W*_0_)/*W*_0_ (8)

where *m_t_* is the moisture content (g-water/g-dray basis), *W_t_* is the sample weight at the ambient absorption time, and *W*_0_ is the initial sample weight. The initial moisture content *m*_0_ (g-water/g-dray basis) was also measured from the initial sample weight W_0_ and the final weights.

## 3. Results and Discussion 

### 3.1. Water Absorption of Soybean under HHP

**Figure 2:**
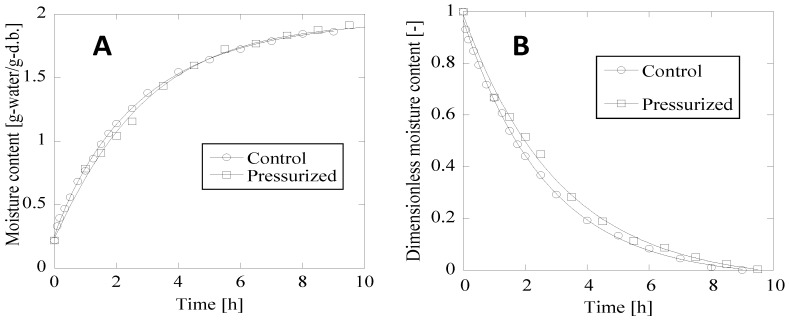
Typical changes in moisture content (**A**) and dimensionless moisture contents (**B**) of untreated control and high hydrostatic pressure (HHP)-treated soybeans. Curves were plotted using Equation (2) for the moisture content, and Equation (3) was used for the dimensionless moisture content.

During water absorption process, soybeans gradually swelled. Changes in the moisture contents of the control and the high-pressure-treated soybeans are shown in Figure 2A, where identical water absorption curves can be seen for the control and the pressure-treated (200 MPa) soybeans. A simple diffusion model represented by Equation (2) was used for measuring water absorption. The model illustrates the homogeneous diffusion of water in the samples under investigation. Therefore, water absorption by the control and the high-pressure-treated soybean sample could be understood as homogeneous water transfer between the testa and the cotyledon. The sorption mechanism was identical in the untreated control and in the HHP-treated soybeans because water could easily permeate through the testa of the bean and reach the cotyledon; hence, the soybean surface remained undamaged even after 10 min of HHP. From the changes in moisture content shown in Figure 2A and Equation (3), the dimensionless moisture content of soybean during water absorption was estimated (Figure 2B). The goodness of fit, determined in terms of the *R*^2^ values of the control and the HHP-treated soybean, were 0.9995 and 0.9985, respectively, confirming the adequacy of the equation for describing the water absorption kinetics of the given materials.

### 3.2. Effective Diffusion Coefficient of Soybean

The effective diffusion coefficient of soybean, Kintoki kidney bean, and toramame bean was estimated from the data fitting curves in Figure 2; the results are listed in Table 1.

**Table 1:** Saturated moisture contents and effective diffusion coefficients of soybean, Kintoki kidney bean, and toramame kidney bean in this study. Effective diffusion coefficients of soybean, green bean, cowpea, brown rice, barley, wheat, and amaranth are cited from references.

Sample	Treatment	Saturated water content (g-water/g-dry basis)	Effective diffusion coefficient (m^2^ s^−1^)	Temperature (°C)	*R*^2^	Reference
Soybean	Untreated	1.9	1.8 × 10^−10^	25	0.999	This study
Soybean	HHP	1.9	1.4 × 10^−10^	25	0.9985	This study
Kintoki kidney bean	Untreated	1.4	3.2 × 10^−10^	25	0.9989	This study
Kintoki kidney bean	HHP	1.5	3.8 × 10^−10^	25	0.9978	This study
Toramame kidney bean	Untreated	1.5	2.2 × 10^−10^	25	0.9995	This study
Soybean	Untreated	-	4.2 × 10^−10^, 2.2 × 10^−10^	25, 40	-	[18,19]
Green bean	Untreated	-	0.8 × 10^−10^–3.6 × 10^−10^	25–55	-	[20]
Green bean	Radiation	-	1.0 × 10^−10^–5.0 × 10^−10^	25–55	-	[20]
Cowpea	Untreated	1.4–1.5	2.4 × 10^−10^–7.9 × 10^−10^	25	-	[21]
Brown rice	Untreated	-	3.9 × 10^−11^	30	-	[15]
Barely	Untreated	-	1.0 × 10^−11^	25	-	[14]
Wheat	Untreated	-	4.0 × 10^−11^	25	-	[14]
Amaranth	Untreated	0.7	2.6 × 10^−11^	25	-	[22]

HHP treatment for 10 min did not influence the water absorption ability of the dried soybean and kintoki kidney bean. Water absorption would occur from the entire surface of the testa in soybean and kintoki kidney bean. Therefore, the rate-limiting step for water sorption in the case of soybean, toramame kidney bean, and green bean would be a testa-wetting process, and the effects of HHP would be insignificant. 

### 3.3. Water Absorption of Adzuki Beans by HHP

**Figure 3:**
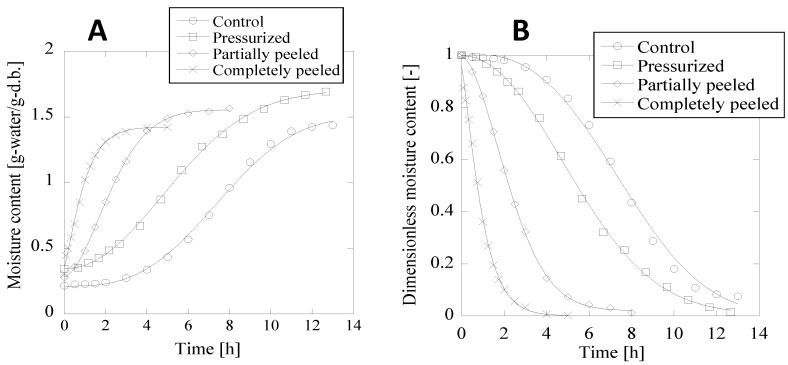
Changes in moisture content (**A**) and dimensionless moisture content (**B**) of adzuki beans, untreated control, HHP (200 MPs)-treated beans, partially peeled (30% peeled) beans, and completely peeled beans. Equations (4) and (5) were used to determine the moisture content and dimensionless moisture content, respectively.

Earlier, we had demonstrated the effect of HHP treatment (200 MPa, at room temperature) on the relative drying rate of Japanese radish [7]. HHP caused in an increase in the drying rate and improved mass transfer from the cells during drying. In the present study, we carried out similar experiments on adzuki beans having a tight testa. Changes in the moisture contents of the control, HHP-treated, partially peeled, and completely peeled adzuki beans are shown in Figure 3A. The water absorption pattern for the HHP-treated adzuki beans was different that for the control. Since the simple diffusion model derived from Equation (2) was unsuitable for data fitting, we applied Weibull-type Equations (4) and (5) for moisture content measurements. The data obtained were used to generate the plots shown in Figure 3, and the *R*^2^ values for the samples were estimated from the data points. The *R*^2^ values for the control, HHP-treated samples, partially peeled samples, and completely peeled samples (0.9981, 0.9995, 0.9998, and 0.9983, respectively) were in close agreement, confirming the adequacy of the Weibull equation in explaining the heterogeneous nature of the water absorption kinetics between the testa and cotyledon of the beans.

Water absorption of adzuki beans were reportedly differed at the moisture content around 60% (dray basis), which may be regarded as a boundary moisture content, while that of soybeans exhibited only the falling rate period. Water absorption could be explained using the theory of diffusion, in the same manner as drying. Tagawa *et al.* earlier had explained the water absorption process based on the Fick’s law of diffusion [9]. Water diffusion is a complex process governed by various parameters such as the microstructure, chemical composition, moisture content, and temperature of the beans. Because of the tight testa, water absorption in the case of the adzuki beans was regulated and significantly slower than that in the other kinds of beans. For this reason, adzuki beans are not frequently used in coarse cereals or consumed widely in Japan.

### 3.4. Effective Diffusion Coefficient of Adzuki Beans

To investigate the influence of HHP on the water uptake of dried beans, we estimated two important parameters: effective diffusion coefficient and water permeation distance. The results are shown in Table 2 and Table 3. The effective diffusion coefficient of the control adzuki bean was 8.6 × 10^−13^ m^2^ s^−1^, while that of the pressure-treated adzuki bean was almost 100 times higher, 6.7 × 10^−11^ m^2^ s^−1^ showing (Table 2). The effective diffusion coefficient of the HHP-treated adzuki beans was significantly larger than that of the control (*p* < 0.05). The completely peeled adzuki beans, on the other hand, gave the maximum value among all samples tested, indicating the absence of the testa and improved mass transfer rate. Further, the average ratio of the testa surfaces in the partially peeled and pressure-treated adzuki beans were similar (31% ± 4.7% and approximately 30%, respectively), suggesting that HHP effectively increased the area for water uptake into the cotyledon. The effective diffusion coefficients (Table 2) for the HHP-treated adzuki beans further supported the role of HHP in increasing water permeability. Although adzuki bean have been attractive for its functional properties, a consumption of adzuki bean was limited because of time-consuming during water uptake. In this study, we showed that HHP could promote a mass transfer in pressurized adzuki bean, which would lead to simultaneous water uptake with cereal coarse. The effective diffusion coefficient is regarded as water uptake ability. If we simultaneously cook several kinds of cereals and legumes in the same cooker, it is reasonable that the effective diffusion coefficient of all kinds of cereals and legumes are similar values because of its homogeneity of quality.

**Table 2:** Saturated moisture contents and effective diffusion coefficients of untreated control, HHP (200 MPa, 10 min)-treated adzuki beans, partially peeled beans, and completely peeled beans. All water absorption experiments were carried out at 25 °C.

Sample	Treatment	Saturated water content (g-water/g-dry basis)		Effective diffusion coefficient (m^2^/s)	Temperature (°C)	*R*^2^
Adzuki bean	Untreated	1.5	8.6 × 10^−13^		25	0.9981
Adzuki bean	HHP	1.7	6.7 × 10^−11^		25	0.9995
Adzuki bean	Partially peeled	1.6	7.3 × 10^−11^		25	0.9998
Adzuki bean	Completely peeled	1.4	4.0 × 10^−10^		25	0.9983

Effective diffusion coefficient of pressurized adzuki bean was 6.7 × 10^−11^ m^2^ s^−1^, while those of brown rice, barely, wheat, and amaranth that we eat as cereal coarse with adzuki beans in Japan were 3.9 × 10^−11^ m^2^ s^−1^, 1.0 × 10^−11^ m^2^ s^−1^, 4.0 × 10^−11^ m^2^ s^−1^, and 2.6 × 10^−11^ m^2^ s^−1^ respectively (Table 1). Therefore, the usage of the untreated control adzuki beans for cereal coarse was altered to pressurized adzuki beans from the viewpoint of cooking time based on the effective diffusion coefficient and homogeneity of quality related to water uptake.

### 3.5. Estimation of Water Permeation Distance

The distance of water permeation into cotyledon during water soaking for 10 min without HHP and the final length of the beans were estimated from the effective diffusion coefficient (Table 1 and Table 2). The permeate lengths in the partially peeled and completely peeled adzuki beans were 0.12 mm and 0.28 mm, respectively, which were nearly tenfold higher than that in the control (0.013 mm). Another experiment with HHP for 10 min decreased the permeate length at the testa surface. The permeate length of the completely peeled adzuki bean and soybean showed similar values. These results confirmed that the testa of the soybeans did not prevent water transfer into the cotyledon.

**Table 3:** Water permeation distance of adzuki bean, soybean, kintoki kidney bean, toramame kidney bean, and white ingen kidney bean during soaking in water for 10 min, and final grain length.

Sample	Treatment	Permeation distance (mm)	Final grain length (mm)
Adzuki bean	Untreated	0.013	7.2
Adzuki bean	Partially peeled	0.12	7.7
Adzuki bean	Completely peeled	0.28	7.7
Soybean	Untreated	0.28	12
Kintoki kidney bean	Untreated	0.099	15
Toramame kidney bean	Untreated	0.051	27
White Ingen kidney bean	Untreated	0.18	17

## 4. Conclusions

The effect of HHP treatment on beans, which were low-moisture-content cellular biological materials, was investigated to study the physical properties of the beans. In the case of adzuki beans, HHP caused an increase in the effective diffusion coefficient, thereby decreasing the water absorption time. The effective diffusion coefficient of HHP-treated adzuki beans was 6.7 × 10^−11^ m^2^ s^−1^, which was similar to that of various other untreated grains: soybean (1.8–4.2 × 10^−10^ m^2^ s^−1^), green bean (0.8–3.6 × 10^−10^ m^2^ s^−1^), brown rice (3.9 × 10^−11^ m^2^ s^−1^), barley (1.0 × 10^−11^ m^2^ s^−1^), wheat (4.0 × 10^−11^ m^2^ s^−1^), and amaranth (2.6 × 10^−12^ m^2^ s^−1^). These results confirmed that HHP treatment causes beneficial structural changes in adzuki beans. The data presented here will serve to increase the demand for adzuki beans for cooking with other foods.
